# Supplementary material for: A Likelihood Approach for Real-Time Calibration of Stochastic Compartmental Epidemic Models
Source: PLoS Comput Biol. 2017 Jan 17;13(1):e1005257. doi: 10.1371/journal.pcbi.1005257 (PMC5240920; doi:10.1371/journal.pcbi.1005257)
Supplement: S1 File — (TAR.GZ) [file pcbi.1005257.s014.tar.gz › HSPH_Online-SI-Revision/IPoi09/n100-severe/n100-severe_BENCH_table4.pdf]

| | | | | | | | | | | | | | | | | | | | | | | | | | | | |
|---|---|---|---|---|---|---|---|---|---|---|---|---|---|---|---|---|---|---|---|---|---|---|---|---|---|---|---|
| 1 | R0 | {1.65278, 9.06793} | {2.76795, 19.6041} | {{2.76795, 19.6041}, {2.76795, 19.6041}} | {67.4724, 116.192} | inf | 4608. | 17154.2 | {11169., 23998.} | 272.27 | 0.553113 | newly | 3152. | 5663.95 | {3553., 8324.} | 80.2379 | 0.234637 | Reff | 0.666979 | 0.531217 | {0.323933, 0.759747} | 42.4598 | ar | 66342. | 92211.6 | {92022., 92408.} | 38.9943 |
| 2 | R0 | {1.4321, 3.80644} | {2.50507, 9.73218} | {{2.50507, 9.73218}, {2.50507, 9.73218}} | {74.9228, 155.677} | inf | 684. | 5036.81 | {2138., 7581.} | 636.375 | 0.783086 | newly | 952. | 2908.79 | {1186., 4325.} | 206.043 | 0.395879 | Reff | 0.675178 | 0.326658 | {0.286455, 0.353791} | 51.619 | ar | 54217. | 89344.6 | {89124., 89559.} | 64.7907 |
| 3 | R0 | {1.68388, 4.76028} | {2.93653, 10.872} | {{2.93653, 10.872}, {2.93653, 10.872}} | {74.3907, 128.39} | inf | 788. | 4929.7 | {2593., 8809.} | 525.597 | 0.736518 | newly | 834. | 2511.43 | {1273., 4569.} | 201.13 | 0.416342 | Reff | 0.549854 | 0.228947 | {0.205293, 0.265844} | 58.3623 | ar | 68299. | 93586.3 | {93426., 93765.} | 37.0244 |
| 4 | R0 | {1.66014, 6.95383} | {2.95209, 14.037} | {{2.95209, 14.037}, {2.95209, 14.037}} | {77.8217, 101.86} | inf | 3183. | 7771.35 | {4342., 14417.} | 144.152 | 0.336487 | newly | 2735. | 3253.58 | {1806., 5989.} | 43.9925 | 0.151864 | Reff | 0.623814 | 0.256662 | {0.216034, 0.322722} | 58.856 | ar | 66820. | 93710.9 | {93555., 93857.} | 40.2438 |
| 5 | R0 | {1.69984, 11.2038} | {2.93978, 19.9817} | {{2.93978, 19.9817}, {2.93978, 19.9817}} | {72.9445, 78.3475} | inf | 5824. | 11947. | {6886., 20407.} | 105.745 | 0.273062 | newly | 3233. | 3768.23 | {2085., 6516.} | 41.3 | 0.149504 | Reff | 0.680072 | 0.33341 | {0.239239, 0.445112} | 56.0565 | ar | 68462. | 93627.3 | {93451., 93834.} | 36.758 |
| 6 | R0 | {1.65876, 6.66217} | {2.81617, 13.9425} | {{2.81617, 13.9425}, {2.81617, 13.9425}} | {69.7756, 109.279} | inf | 3387. | 10061.3 | {5720., 15478.} | 197.057 | 0.426323 | newly | 2924. | 4314.21 | {2357., 6787.} | 60.099 | 0.180154 | Reff | 0.632386 | 0.31986 | {0.252948, 0.376156} | 50.7731 | ar | 67105. | 92631.6 | {92473., 92787.} | 38.0398 |
| 7 | R0 | {1.69966, 5.89996} | {2.79531, 12.1249} | {{2.79531, 12.1249}, {2.79531, 12.1249}} | {64.4629, 105.508} | inf | 2191. | 9094.77 | {5080., 13219.} | 315.097 | 0.57543 | newly | 2014. | 4364.77 | {2349., 6239.} | 117.395 | 0.291153 | Reff | 0.564848 | 0.308841 | {0.253087, 0.354557} | 47.0127 | ar | 69396. | 92457.4 | {92317., 92603.} | 33.2315 |
| 8 | R0 | {1.53428, 6.3734} | {2.8833, 17.1847} | {{2.8833, 17.1847}, {2.8833, 17.1847}} | {87.9253, 169.632} | inf | 2878. | 14866.2 | {8655., 25219.} | 416.547 | 0.68484 | newly | 2677. | 5449.66 | {3048., 9469.} | 104.171 | 0.27699 | Reff | 0.700629 | 0.411702 | {0.272501, 0.615325} | 51.4512 | ar | 59855. | 93197.4 | {93009., 93386.} | 55.7052 |
| 9 | R0 | {1.58939, 3.48608} | {2.96796, 12.4643} | {{2.96796, 12.4643}, {2.96796, 12.4643}} | {86.7358, 257.545} | inf | 666. | 10993.9 | {6310., 18367.} | 1550.73 | 1.17301 | newly | 876. | 5169.92 | {2804., 8757.} | 490.173 | 0.718842 | Reff | 0.606066 | 0.304088 | {0.232302, 0.38815} | 53.1466 | ar | 62575. | 93827.4 | {93696., 93953.} | 49.9438 |
| 10 | R0 | {1.58528, 5.5593} | {2.93978, 19.9817} | {{2.93978, 19.9817}, {2.93978, 19.9817}} | {85.4423, 259.428} | inf | 2018. | 17255.9 | {11429., 24300.} | 755.099 | 0.913147 | newly | 2025. | 5572.89 | {3524., 8114.} | 175.204 | 0.415798 | Reff | 0.636823 | 0.476888 | {0.287716, 0.732946} | 49.6203 | ar | 63039. | 93627.3 | {93451., 93834.} | 48.5228 |
| 11 | R0 | {1.58305, 4.62721} | {2.9884, 12.8326} | {{2.9884, 12.8326}, {2.9884, 12.8326}} | {88.7748, 177.329} | inf | 1344. | 8401.02 | {4831., 13395.} | 525.076 | 0.751975 | newly | 1559. | 3791.1 | {2115., 6056.} | 143.175 | 0.337365 | Reff | 0.61568 | 0.256968 | {0.215015, 0.305146} | 59.084 | ar | 63222. | 93983.5 | {93851., 94123.} | 48.6563 |
| 12 | R0 | {1.70234, 9.34626} | {2.76795, 19.6041} | {{2.76795, 19.6041}, {2.76795, 19.6041}} | {62.5968, 109.753} | inf | 5033. | 17154.2 | {11169., 23998.} | 240.835 | 0.514798 | newly | 3340. | 5663.95 | {3553., 8324.} | 70.8653 | 0.212925 | Reff | 0.662312 | 0.531217 | {0.323933, 0.759747} | 42.2623 | ar | 68213. | 92211.6 | {92022., 92408.} | 35.1818 |
| 13 | R0 | {1.68556, 9.74954} | {2.76795, 19.6041} | {{2.76795, 19.6041}, {2.76795, 19.6041}} | {64.2155, 101.077} | inf | 4862. | 14619.4 | {8764., 22539.} | 200.686 | 0.450609 | newly | 3072. | 4780.26 | {2785., 7415.} | 60.5768 | 0.182922 | Reff | 0.650053 | 0.444605 | {0.293375, 0.599344} | 44.9538 | ar | 68426. | 92211.6 | {92022., 92408.} | 34.761 |
| 14 | R0 | {1.59876, 9.78897} | {2.78751, 19.9497} | {{2.79269, 19.984}, {2.79269, 19.984}} | {74.3547, 103.797} | inf | 5531. | 14425.9 | {8866., 21501.} | 161.74 | 0.396287 | newly | 3640. | 4638.93 | {2768., 7043.} | 38.348 | 0.133205 | Reff | 0.761314 | 0.477577 | {0.298036, 0.559151} | 55.9449 | ar | 63553. | 92376.3 | {92107., 92594.} | 45.3532 |
| 15 | R0 | {1.43577, 5.12819} | {2.73737, 17.9172} | {{2.73737, 17.9172}, {2.73737, 17.9172}} | {90.6552, 249.386} | inf | 2030. | 15187.2 | {9022., 22787.} | 648.14 | 0.848603 | newly | 2303. | 5422.81 | {3133., 8140.} | 135.467 | 0.340777 | Reff | 0.736378 | 0.483043 | {0.306175, 0.719764} | 48.2637 | ar | 53259. | 91915.1 | {91759., 92090.} | 72.5813 |
| 16 | R0 | {1.58866, 8.73191} | {2.76795, 19.6041} | {{2.76795, 19.6041}, {2.76795, 19.6041}} | {74.2317, 124.511} | inf | 5612. | 17154.2 | {11169., 23998.} | 205.67 | 0.467507 | newly | 4036. | 5663.95 | {3553., 8324.} | 46.0905 | 0.152785 | Reff | 0.723984 | 0.531217 | {0.323933, 0.759747} | 44.8193 | ar | 64349. | 92211.6 | {92022., 92408.} | 43.2992 |
| 17 | R0 | {1.43012, 3.8163} | {2.87181, 13.004} | {{2.87181, 13.004}, {2.87181, 13.004}} | {100.809, 240.749} | inf | 1091. | 12162.5 | {6712., 19561.} | 1014.8 | 1.00974 | newly | 1516. | 5548.75 | {2972., 8957.} | 266.013 | 0.52041 | Reff | 0.70422 | 0.341373 | {0.254643, 0.435137} | 53.6092 | ar | 53353. | 93092.1 | {92926., 93258.} | 74.4834 |
| 18 | R0 | {1.47965, 5.16472} | {2.86193, 14.6465} | {{2.86193, 14.6465}, {2.86193, 14.6465}} | {93.4194, 183.587} | inf | 1756. | 12212.3 | {7493., 19529.} | 595.461 | 0.806793 | newly | 1958. | 5031.02 | {3009., 8177.} | 156.947 | 0.369489 | Reff | 0.705823 | 0.339637 | {0.259835, 0.443599} | 52.5995 | ar | 56086. | 93021.9 | {92830., 93201.} | 65.8558 |
| 19 | R0 | {1.57191, 5.2314} | {2.86001, 13.5542} | {{2.86001, 13.5542}, {2.86001, 13.5542}} | {81.9449, 159.093} | inf | 1293. | 8055.79 | {3986., 12623.} | 523.031 | 0.736357 | newly | 1298. | 3507.1 | {1659., 5541.} | 170.192 | 0.36881 | Reff | 0.610687 | 0.281902 | {0.229001, 0.322924} | 54.5949 | ar | 63119. | 92998.7 | {92780., 93186.} | 47.3386 |
| 20 | R0 | {1.54435, 5.32911} | {2.8833, 17.1847} | {{2.8833, 17.1847}, {2.8833, 17.1847}} | {86.6999, 222.468} | inf | 1762. | 14866.2 | {8655., 25219.} | 743.712 | 0.897925 | newly | 1849. | 5449.66 | {3048., 9469.} | 194.735 | 0.434866 | Reff | 0.632813 | 0.411702 | {0.272501, 0.615325} | 47.6626 | ar | 61870. | 93197.4 | {93009., 93386.} | 50.6342 |
| 21 | R0 | {1.57932, 4.90031} | {2.79531, 12.1249} | {{2.79531, 12.1249}, {2.79531, 12.1249}} | {76.9945, 147.431} | inf | 1650. | 6154.53 | {3310., 8957.} | 273.002 | 0.519994 | newly | 1742. | 2912.12 | {1543., 4259.} | 72.6607 | 0.195884 | Reff | 0.608496 | 0.269021 | {0.236847, 0.299294} | 55.7893 | ar | 63595. | 92457.4 | {92317., 92603.} | 45.3846 |
| 22 | R0 | {1.47044, 4.07694} | {2.94354, 15.7922} | {{2.96947, 15.963}, {2.96947, 15.963}} | {100.181, 287.355} | inf | 1362. | 11270.9 | {5955., 20482.} | 727.523 | 0.875307 | newly | 1880. | 4359.8 | {2217., 8009.} | 131.904 | 0.316896 | Reff | 0.691357 | 0.323221 | {0.229659, 0.437937} | 58.3068 | ar | 55895. | 93653.6 | {93646., 93970.} | 67.5528 |
| 23 | R0 | {1.64997, 3.34546} | {2.82466, 9.2843} | {{2.82466, 9.2843}, {2.82466, 9.2843}} | {71.1946, 177.519} | inf | 230. | 3402.62 | {1736., 4871.} | 1379.4 | 1.09868 | newly | 308. | 1961.12 | {988., 2840.} | 536.726 | 0.729748 | Reff | 0.558729 | 0.235451 | {0.21764, 0.253428} | 57.8596 | ar | 66404. | 92686.6 | {92521., 92865.} | 39.5798 |
| 24 | R0 | {1.67405, 4.50254} | {2.98388, 9.60029} | {{2.98388, 9.60029}, {2.98388, 9.60029}} | {78.2432, 113.219} | inf | 1189. | 4898.45 | {2711., 7871.} | 311.981 | 0.560876 | newly | 1389. | 2734.71 | {1467., 4458.} | 98.595 | 0.244797 | Reff | 0.560623 | 0.219218 | {0.196697, 0.240023} | 60.8973 | ar | 68017. | 93948. | {93795., 94112.} | 38.1243 |
| 25 | R0 | {1.50663, 3.1286} | {2.7566, 8.57964} | {{2.7566, 8.57964}, {2.7566, 8.57964}} | {82.9646, 174.233} | inf | 450. | 3905.85 | {1880., 6012.} | 767.967 | 0.88083 | newly | 698. | 2394.78 | {1109., 3607.} | 243.092 | 0.475268 | Reff | 0.634201 | 0.252376 | {0.231995, 0.274034} | 60.2057 | ar | 58823. | 92098. | {91922., 92232.} | 56.568 |
| 26 | R0 | {1.69822, 6.34992} | {2.8939, 16.8013} | {{2.8939, 16.8013}, {2.8939, 16.8013}} | {70.4078, 164.591} | inf | 2723. | 16686.4 | {10283., 25758.} | 512.794 | 0.763967 | newly | 2372. | 6275.62 | {3859., 10869.} | 164.571 | 0.392902 | Reff | 0.56493 | 0.452107 | {0.29376, 0.732301} | 42.0355 | ar | 69816. | 93276.2 | {93112., 93387.} | 33.6029 |
| 27 | R0 | {1.55869, 4.92988} | {2.99911, 15.6207} | {{2.99911, 15.6207}, {2.99911, 15.6207}} | {92.4122, 216.858} | inf | 1548. | 13170.9 | {7358., 22520.} | 750.835 | 0.890838 | newly | 1681. | 5172.58 | {2817., 8955.} | 207.709 | 0.442862 | Reff | 0.625643 | 0.337431 | {0.233871, 0.488525} | 52.5415 | ar | 62344. | 94013.6 | {93846., 94150.} | 50.7982 |
| 28 | R0 | {1.46085, 6.06899} | {2.44101, 12.0941} | {{2.44101, 12.0941}, {2.44101, 12.0941}} | {67.0952, 99.277} | inf | 2116. | 8091.64 | {3538., 14867.} | 282.403 | 0.526399 | newly | 2108. | 4051.46 | {1704., 7507.} | 99.4114 | 0.255279 | Reff | 0.722157 | 0.413118 | {0.31799, 0.505216} | 48.5969 | ar | 55484. | 88460.4 | {88209., 88708.} | 59.4341 |
| 29 | R0 | {1.63017, 6.25164} | {2.99911, 15.6207} | {{2.99911, 15.6207}, {2.99911, 15.6207}} | {83.9753, 149.866} | inf | 2357. | 13170.9 | {7358., 22520.} | 458.8 | 0.708249 | newly | 2132. | 5172.58 | {2817., 8955.} | 142.777 | 0.340357 | Reff | 0.606896 | 0.337431 | {0.233871, 0.488525} | 51.5738 | ar | 66231. | 94013.6 | {93846., 94150.} | 41.9481 |
| 30 | R0 | {1.45173, 5.68165} | {2.93978, 19.9817} | {{2.93978, 19.9817}, {2.93978, 19.9817}} | {102.502, 251.688} | inf | 2119. | 17255.9 | {11429., 24300.} | 714.342 | 0.891937 | newly | 2202. | 5572.89 | {3524., 8114.} | 153.083 | 0.379405 | Reff | 0.721074 | 0.476888 | {0.287716, 0.732946} | 52.8251 | ar | 55280. | 93627.3 | {93451., 93834.} | 69.3692 |
| 31 | R0 | {1.55808, 2.77316} | {2.78624, 7.73801} | {{2.78624, 7.73801}, {2.78624, 7.73801}} | {78.8252, 179.032} | inf | 226. | 1420.41 | {700., 2052.} | 528.499 | 0.734926 | newly | 342. | 927.831 | {454., 1329.} | 171.295 | 0.367969 | Reff | 0.613478 | 0.223742 | {0.21663, 0.230729} | 63.529 | ar | 60940. | 92376.6 | {92163., 92571.} | 51.5862 |
| 32 | R0 | {1.56797, 3.90327} | {2.93597, 11.6547} | {{2.93597, 11.6547}, {2.93597, 11.6547}} | {87.2466, 198.588} | inf | 914. | 7795.36 | {4264., 14468.} | 752.884 | 0.886868 | newly | 1187. | 3790.05 | {2034., 7133.} | 219.296 | 0.457155 | Reff | 0.61673 | 0.25845 | {0.22099, 0.336961} | 58.0935 | ar | 62228. | 93584.1 | {93441., 93733.} | 50.389 |
| 33 | R0 | {1.70027, 5.43261} | {2.93653, 10.872} | {{2.93653, 10.872}, {2.93653, 10.872}} | {72.7096, 100.125} | inf | 1215. | 4929.7 | {2593., 8809.} | 305.737 | 0.548468 | newly | 1142. | 2511.43 | {1273., 4569.} | 121.169 | 0.285615 | Reff | 0.555682 | 0.228947 | {0.205293, 0.265844} | 58.799 | ar | 68798. | 93586.3 | {93426., 93765.} | 36.0306 |
| 34 | R0 | {1.61668, 6.86192} | {2.7811, 19.6712} | {{2.7811, 19.6712}, {2.7811, 19.6712}} | {72.0254, 186.672} | inf | 2934. | 16386.8 | {11288., 22247.} | 458.515 | 0.731168 | newly | 2525. | 5375.68 | {3553., 7361.} | 112.898 | 0.309047 | Reff | 0.646575 | 0.506813 | {0.32486, 0.666101} | 44.5514 | ar | 64693. | 92343.4 | {92163., 92489.} | 42.7409 |
| 35 | R0 | {1.55955, 3.45071} | {2.93906, 10.3908} | {{2.93906, 10.3908}, {2.93906, 10.3908}} | {88.4556, 201.121} | inf | 514. | 5685.62 | {3202., 8776.} | 1006.15 | 0.992918 | newly | 674. | 3005.05 | {1694., 4679.} | 345.853 | 0.595858 | Reff | 0.608474 | 0.235665 | {0.211612, 0.254317} | 61.2694 | ar | 61783. | 93608.6 | {93494., 93734.} | 51.5118 |
| 36 | R0 | {1.65068, 4.91307} | {2.99939, 13.7076} | {{2.99939, 13.7076}, {2.99939, 13.7076}} | {81.7063, 179.003} | inf | 1866. | 12170. | {7273., 20093.} | 552.2 | 0.78214 | newly | 2044. | 5277.76 | {3127., 8770.} | 158.207 | 0.37441 | Reff | 0.596341 | 0.311115 | {0.231703, 0.402248} | 52.2304 | ar | 66453. | 94036.2 | {93909., 94200.} | 41.5079 |
| 37 | R0 | {1.46131, 3.06714} | {2.54659, 8.24572} | {{2.54659, 8.24572}, {2.54659, 8.24572}} | {74.2676, 168.841} | inf | 344. | 3280.11 | {1444., 6209.} | 853.521 | 0.90037 | newly | 544. | 2102.95 | {903., 4017.} | 286.572 | 0.50481 | Reff | 0.646659 | 0.292148 | {0.269888, 0.324028} | 54.8219 | ar | 56452. | 89847. | {89663., 90069.} | 59.1564 |
| 38 | R0 | {1.71034, 5.2546} | {2.9884, 12.8326} | {{2.9884, 12.8326}, {2.9884, 12.8326}} | {74.7255, 144.216} | inf | 1519. | 8401.02 | {4831., 13395.} | 453.062 | 0.698817 | newly | 1497. | 3791.1 | {2115., 6056.} | 153.246 | 0.354989 | Reff | 0.547719 | 0.256968 | {0.215015, 0.305146} | 54.4075 | ar | 69816. | 93983.5 | {93851., 94123.} | 34.616 |
| 39 | R0 | {1.43565, 5.57989} | {2.4982, 12.6322} | {{2.4534, 12.3139}, {2.63407, 13.6136}} | {74.0115, 126.389} | inf | 1796. | 6568.47 | {2919., 12507.} | 265.728 | 0.494298 | newly | 1875. | 3062.16 | {1397., 5981.} | 77.8894 | 0.217839 | Reff | 0.709096 | 0.347175 | {0.284427, 0.448776} | 51.0398 | ar | 54407. | 89221.1 | {88405., 90904.} | 63.9883 |
| 40 | R0 | {1.40327, 4.00764} | {2.28593, 9.04943} | {{2.28593, 9.04943}, {2.28593, 9.04943}} | {62.9002, 125.804} | inf | 1177. | 6539.02 | {2665., 13111.} | 455.567 | 0.666025 | newly | 1644. | 4211.09 | {1584., 8407.} | 160.591 | 0.337937 | Reff | 0.717576 | 0.440286 | {0.346798, 0.533399} | 44.954 | ar | 51976. | 86019.3 | {85751., 86262.} | 65.4982 |
| 41 | R0 | {1.63612, 4.26741} | {2.82914, 9.34414} | {{2.82914, 9.34414}, {2.82914, 9.34414}} | {72.9176, 118.965} | inf | 805. | 3375.02 | {1627., 4456.} | 319.257 | 0.560593 | newly | 987. | 1931.02 | {903., 2529.} | 98.1138 | 0.238282 | Reff | 0.590394 | 0.232934 | {0.215694, 0.239713} | 60.5459 | ar | 65029. | 92759.3 | {92621., 92895.} | 42.6429 |
| 42 | R0 | {1.57426, 6.50271} | {2.97157, 19.3547} | {{2.97157, 19.3547}, {2.97157, 19.3547}} | {88.7598, 197.641} | inf | 2814. | 17815.3 | {10829., 26177.} | 533.095 | 0.780182 | newly | 2604. | 5928.95 | {3499., 9374.} | 127.686 | 0.331 | Reff | 0.666321 | 0.482094 | {0.281437, 0.75888} | 49.9428 | ar | 62522. | 93849.1 | {93703., 93993.} | 50.1057 |
| 43 | R0 | {1.37818, 5.37636} | {2.48143, 15.7026} | {{2.48143, 15.7026}, {2.48143, 15.7026}} | {80.0512, 192.067} | inf | 1811. | 12793.3 | {8359., 20215.} | 606.42 | 0.823831 | newly | 2047. | 5162.03 | {3229., 8281.} | 152.175 | 0.371727 | Reff | 0.756841 | 0.522194 | {0.367351, 0.741997} | 43.1457 | ar | 50098. | 89043. | {88838., 89242.} | 77.7377 |
| 44 | R0 | {1.66606, 4.92641} | {2.96796, 12.4643} | {{2.96796, 12.4643}, {2.96796, 12.4643}} | {78.1424, 153.01} | inf | 1739. | 10993.9 | {6310., 18367.} | 532.196 | 0.756189 | newly | 1807. | 5169.92 | {2804., 8757.} | 186.105 | 0.404388 | Reff | 0.583371 | 0.304088 | {0.232302, 0.38815} | 51.783 | ar | 67066. | 93827.4 | {93696., 93953.} | 39.903 |
| 45 | R0 | {1.623, 5.9449} | {2.86001, 13.5542} | {{2.86001, 13.5542}, {2.86001, 13.5542}} | {76.2175, 127.997} | inf | 2642. | 11178.3 | {5917., 17434.} | 323.102 | 0.581545 | newly | 2549. | 4929.11 | {2527., 7571.} | 95.5752 | 0.245382 | Reff | 0.641799 | 0.33357 | {0.246018, 0.401631} | 51.6802 | ar | 64554. | 92998.7 | {92780., 93186.} | 44.0634 |
| 46 | R0 | {1.65235, 5.51234} | {2.96947, 15.963} | {{2.96947, 15.963}, {2.96947, 15.963}} | {79.7119, 189.587} | inf | 2356. | 14633.1 | {8637., 21308.} | 521.1 | 0.766486 | newly | 2372. | 5688.23 | {3225., 9960.} | 139.807 | 0.346419 | Reff | 0.608825 | 0.390185 | {0.252969, 0.590271} | 51.2358 | ar | 66509. | 93829. | {93704., 93974.} | 41.0771 |
| 47 | R0 | {1.62138, 8.82208} | {2.96871, 19.4111} | {{2.97157, 19.3547}, {2.97157, 19.3547}} | {83.0977, 120.029} | inf | 4208. | 15081.1 | {8267., 25618.} | 258.391 | 0.521233 | newly | 2927. | 4932.89 | {2610., 8636.} | 72.2721 | 0.205259 | Reff | 0.666095 | 0.398126 | {0.254218, 0.598833} | 52.1982 | ar | 65598. | 93835. | {93670., 93993.} | 43.0454 |
| 48 | R0 | {1.64073, 6.69261} | {2.86001, 13.5542} | {{2.86001, 13.5542}, {2.86001, 13.5542}} | {74.3133, 102.525} | inf | 3295. | 11178.3 | {5917., 17434.} | 239.252 | 0.485622 | newly | 2847. | 4929.11 | {2527., 7571.} | 78.1939 | 0.21148 | Reff | 0.626628 | 0.33357 | {0.246018, 0.401631} | 50.7485 | ar | 66438. | 92998.7 | {92780., 93186.} | 39.9781 |
| 49 | R0 | {1.65556, 3.796} | {2.98388, 9.60029} | {{2.98388, 9.60029}, {2.98388, 9.60029}} | {80.2339, 152.905} | inf | 539. | 4898.45 | {2711., 7871.} | 808.804 | 0.904469 | newly | 685. | 2734.71 | {1467., 4458.} | 299.228 | 0.543688 | Reff | 0.564513 | 0.219218 | {0.196697, 0.240023} | 61.1668 | ar | 66575. | 93948. | {93795., 94112.} | 41.116 |
| 50 | R0 | {1.56828, 3.65713} | {2.82466, 9.2843} | {{2.82466, 9.2843}, {2.82466, 9.2843}} | {80.112, 153.868} | inf | 506. | 3402.62 | {1736., 4871.} | 572.454 | 0.756259 | newly | 685. | 1961.12 | {988., 2840.} | 186.436 | 0.383229 | Reff | 0.595868 | 0.235451 | {0.21764, 0.253428} | 60.4861 | ar | 62761. | 92686.6 | {92521., 92865.} | 47.6818 |
